# Impact of Scribes with Flow Coordination Duties on Throughput in an Academic Emergency Department

**DOI:** 10.5811/westjem.2020.2.46110

**Published:** 2020-04-24

**Authors:** Keith Thomas, Joshua Marcum, Alexei Wagner, Michael A. Kohn

**Affiliations:** * Stanford Hospital and Clinics, Department of Emergency Medicine, Stanford, California; † Stanford University School of Medicine, Department of Emergency Medicine, Stanford, California

## Abstract

**Introduction:**

With the increasing influence of electronic health records in emergency medicine came concerns of decreasing operational efficiencies. Particularly worrisome was increasing patient length of stay (LOS). Medical scribes were identified to be in a good position to quickly address barriers to treatment delivery and patient flow. The objective of this study was to investigate patient LOS in the mid- and low-acuity zones of an academic emergency department (ED) with and without medical scribes.

**Methods:**

A retrospective cohort study compared patient volume and average LOS between a cohort without scribes and a cohort after the implementation of a scribe-flow coordinator program. Patients were triaged to the mid-acuity Vertical Zone (primarily Emergency Severity Index [ESI] 3) or low-acuity Fast Track (primarily ESI 4 and 5) at a tertiary academic ED. Patients were stratified by treatment zone, acuity level, and disposition.

**Results:**

The pre-intervention and post-intervention periods included 8900 patients and 9935 patients, respectively. LOS for patients discharged from the Vertical Zone decreased by 12 minutes from 235 to 223 minutes (p<0.0001, 95% confidence interval [CI], −17,−7) despite a 10% increase in patient volume. For patients admitted from the Vertical Zone, volume increased 13% and LOS remained almost the same, increasing from 225 to 228 minutes (p=0.532, 95% CI, −6,12). For patients discharged from the Fast Track, volume increased 14% and LOS increased six minutes, from 89 to 95 minutes (p<0.0001, 95% CI, 4,9). Predictably, only 1% of Fast Track patients were admitted.

**Conclusion:**

Despite substantially increased volume, the use of scribes as patient flow facilitators in the mid-acuity zone was associated with decreased LOS. In the low-acuity zone, scribes were not shown to be as effective, perhaps because rapid patient turnover required them to focus on documentation.

## INTRODUCTION

### Background

The advent of electronic health records promised an improvement in healthcare quality, safety, outcomes, and clinic-related efficiencies. 1–3 Evidence suggests electronic health records have important structural- and process-related benefits. 4–8 However, over the short term, they may reduce productivity, potentially increasing provider documentation time and patient length of stay (LOS). 9–16 To help address these increasing concerns about physician efficiency and rapid patient throughput 17,18, as well as the issue of worsening emergency department (ED) crowding 19–23, the use of medical scribes increased nationwide. 24–26

### Importance

**Figure 1:**
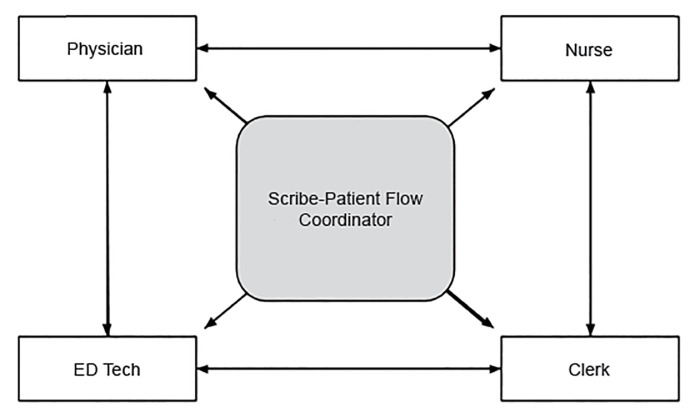
Representation of emergency department (ED) staff communication associations using a scribe as a patient flow coordinator.

Strategies that reduce LOS and improve patient flow are critical to the efficient and humane delivery of emergency medical care. 27 Implementation of traditional scribe programs has been shown to be beneficial. 1, 28, 29 At various institutions, scribes have been tasked with ancillary responsibilities to facilitate patient throughput 30, 31 since they may be in a good position to quickly address potential barriers to treatment delivery and patient flow (Figure 1). However, the full effectiveness of these programs remains unknown.

### Goals of this Investigation

In addition to the traditional documentation role, scribes were tasked with facilitating throughput in mid- and low-acuity treatment zones of an academic ED. This study assessed the impact of scribe-flow coordinators on patient throughput by comparing LOS between a pre-implementation period without any scribes and a post-implementation period with scribe-flow coordinators in both treatment zones.

## METHODS

### Study Design

The study used a retrospective cohort design and was approved by our institutional review board (IRB) with waiver of informed consent.

### Study Setting and Population

The setting for the study was the ED of a level I trauma and tertiary care center. The ED has seen a 5–8% annual increase in patient volume over the past several years and had 71,500 patient visits in 2016.

Emergency Severity Index (ESI) 3 patients aged 14 years and older who were capable of sitting in reclining chairs were seen in a mid-acuity Vertical Zone. It was operational 13 hours per day and staffed by one attending physician, one senior resident, one first-year resident, four to one nursing staff plus a throughput nurse, one ED technician, and one clerk. The Vertical Zone had the baseline capacity to treat 12 patients and could flex to 16 patients if needed. ESI 4 and 5 patients aged six months and older were seen in a low-acuity Fast Track. This zone was operational 12 hours per day and staffed by one attending, one to two nurses, and one ED technician. The Fast Track was capable of treating up to 10 patients at a time. These zones were established prior to the development of the scribe-flow coordinator program and were not part of this study. Prior to this cohort, there was no scribe utilization in any area within this ED.

**Population Health Research Capsule**

What do we already know about this issue?

By alleviating a physician’s documentation burden, medical scribes can help address concerns about physician efficiency and patient throughput.

What was the research question?

If scribes were also tasked with facilitating throughput, what effect can they have on patient length of stay (LOS)?

What was the major finding of the study?

Using scribes as patient flow facilitators in the mid-acuity zone was associated with decreased LOS of 12 minutes per patient. In the low-acuity zone, scribes were less effective (no change), perhaps because rapid patient turnover required focus on documentation.

How does this improve population health?

Tasking medical scribes with additional flow coordination responsibilities can be a solution for departments looking to improve patient LOS.

### Study Protocol

Patients were triaged to either the Vertical Zone or Fast Track if they arrived during operational hours and met the appropriate clinical criteria. We evaluated all patient encounters in the Vertical Zone and Fast Track during two six-month periods before any scribe implementation and after scribe-flow coordinator implementation for study inclusion. The pre-intervention period without scribes was July 1–December 31, 2014, and the post-intervention period with scribes was July 1–December 31, 2015. July through December of both years were used to control for seasonal variations.

**Figure 2:**
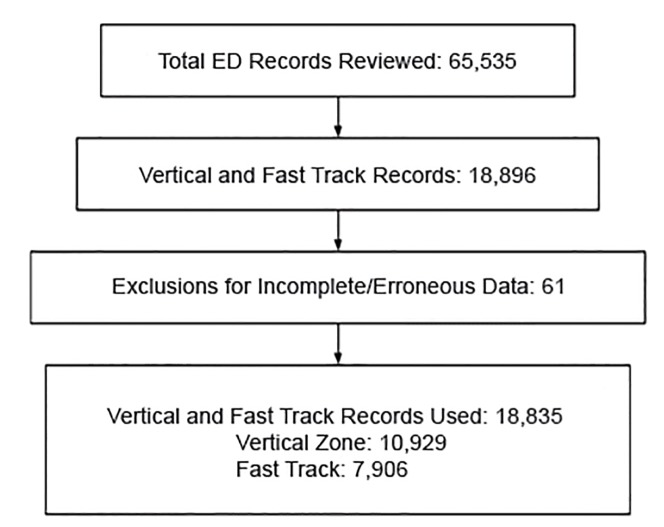
Process of emergency department (ED) record selection for 2014 and 2015.

Patients were excluded if their LOS could not be determined because of missing or incomplete data. Intradepartmental room transfers did not allow a full complement of the scribe-flow coordinator’s intervention so these patients were excluded from the data set. We also excluded conspicuously erroneous data (eg, LOS values less than 0 minutes or greater than 13 hours). After removing patient charts with any such data, a total of 10,929 Vertical Zone encounters and 7,906 Fast Track encounters were examined in this study (Figure 2).

A group of scribe-flow coordinators with a wide variety of medical backgrounds (eg, emergency medical service, foreign medical school graduation) was interviewed from outside applications and hired in April 2015 and trained over a two-month period using an in-house curriculum developed by emergency medicine faculty and nursing supervisors to ensure competency and uniformity of desired performance. A customized curriculum was necessary in order to outline institutional flow coordinating protocol (eg, how often to check for lab updates, how to address consultant delays, etc). Their schedules were developed separately from those of attending physicians.

**Figure 3:**
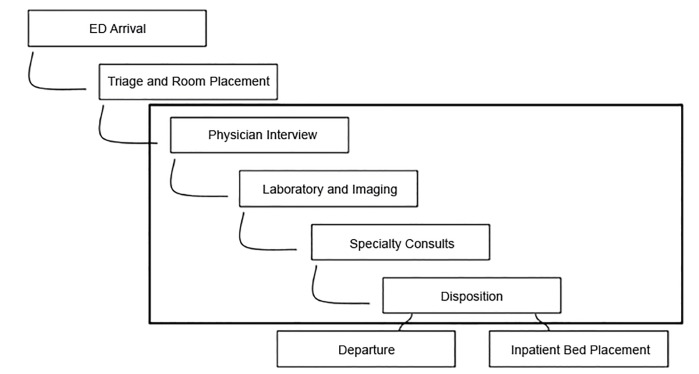
Representation of a scribe-patient flow coordinator intervention in emergency department (ED) flow.

Since institutional policy prohibited first-year residents from using documentation assistance, not all patient encounters were documented by the scribe-flow coordinator in the Vertical Zone. However, the scribe-flow coordinator was still responsible for facilitating throughput for all active patients within the Vertical Zone. Specific functions included ensuring closed-loop communication between staff, mitigating consultant delays, taking a proactive role in tracking lab and imaging results, updating patients, and completing non-clinical discharge tasks (Figure 3). These tasks were completed as expected by every scribe-flow coordinator on each shift. The intent was to have the scribe-flow coordinator share a holistic view of all patients so that treatment and disposition would be maximally efficient.

### Measures

Data on ED patients triaged to the Vertical Zone and the Fast Track during the two study periods were extracted from the hospital’s electronic database and managed in accordance with IRB protocol. We examined throughput metrics for each patient encounter.

The outcome variable was LOS in minutes. For admitted patients, LOS was arrival time to admit order time. For discharged patients, LOS was arrival time to discharge order time. When compared to after visit summary print time or nursing discharge timestamps, the discharge order time metric reflected the scribes’ contributions most accurately because it was the least dependent on variable nursing workload and constraints.We also measured daily patient volume and the patient demographics of age and gender.

### Data Analysis

We compared patient demographics of age and gender. The comparison of LOS between periods was stratified by treatment zone, ESI acuity level, and disposition. We compared categorical variables using the chi-square test. Continuous variables, including LOS, were compared using the t-test and reporting 95% confidence intervals (CI). We performed statistical analysis using Stata 13 (Statacorp, College Station, TX).

## RESULTS

### Characteristics of Study Subjects

In the Vertical Zone, 5201 patients with complete data were seen in 2014 without a scribe-flow coordinator present and 5728 were seen in 2015 with scribe-flow coordinators. The Fast Track saw 3699 in 2014 without scribe-flow coordinators and 4207 in 2015 with scribe-flow coordinators.

In the Fast Track, the proportion of patients aged 18–64 years decreased significantly by 3.3% (95% confidence interval [CI], 0.02–0.08). Otherwise the demographic characteristics did not differ between the two time periods (Table 1).

### Main Results–Vertical Zone

In the pre-intervention cohort (2014), the Vertical Zone volume was 28.3 patients per day (2.2 patients per hour). In the post-intervention cohort (2015), daily volume increased by 2.9 to 31.1 patients per day and hourly volume increased to 2.4 patients per hour. LOS for patients treated and discharged decreased by 12 minutes from 235 minutes to 223 minutes (p<0.0001, 95% CI, −17,−7) despite the 10% increase in volume. For patients admitted from the Vertical Zone, volume increased by 13% and LOS remained almost the same, increasing by three minutes from 225 minutes to 228 minutes, which was not statistically significant (p = 0.532, 95% CI, 6,12) (Table 2).

### Main Results–Fast Track

In the pre-intervention cohort, the Fast Track volume was 20.1 patients per day (1.7 patients per hour). In the post-intervention cohort, the Fast Track daily volume increased by 2.9 to 23 patients per day (1.9 patients per hour) in 2015. For patients discharged, patient volume increased by 14% with a corresponding increase in LOS of 6 minutes, from 89 minutes to 95 minutes (p<0.0001, 95% CI, 4,9). Predictably, only 1% of Fast Track patients were admitted, 30 in the pre-intervention period and 54 in the post-intervention period with an insignificant 27-minute increase in LOS.

## DISCUSSION

In the medium-acuity Vertical Zone, scribe-flow coordinators helped enable clinical providers to treat 9.7% more discharged patients while decreasing LOS by 5% and to treat 13% more admitted patients with no change in total LOS. In the low-acuity Fast Track, volume increased by 13.6%, but LOS also increased by 7%.

Our literature review failed to identify any published studies that stratified throughput metrics on ESI-specific treatment zones. Studies by Allen et al and Bastani et al also showed improvements in throughput times after implementation of a scribe program. 28, 32 However, other studies have shown no significant improvement in LOS with most, including a meta-analysis by Heaton et al, also citing an increase in patient volume. 29, 33–36 Therefore, implementation expectations and details are of great consequence. 36 By the end of 2015, the Vertical Zone and Fast Track accounted for approximately one-third of the total ED census. Patient volume growths of 10% in the Vertical Zone and 14% in the Fast Track were significantly higher than the 5% annual increase for the entire ED. As with nurse-flow coordinators who decreased LOS by maintaining open communication with inpatient units, 20 scribe-flow coordinators appear to have decreased LOS in the Vertical Zone by facilitating communication between ED staff and patients.

Since patients generally require a more involved medical workup in the Vertical Zone than in the Fast Track, scribe-flow coordinators have more opportunities to decrease LOS for patients seen in the Vertical Zone. Ensuring optimal staff communication, mitigating consultant delays, and taking a proactive role in tracking lab and imaging results are a few such possible interventions. In contrast, the typical Fast Track patient’s ED stay often involves immediate disposition with no need for laboratory or imaging studies, so the impact of the scribe’s flow coordination duties on throughput in this low-acuity zone may be minimal.

## LIMITATIONS

Concurrent to this study, various protocol changes and new initiatives had been implemented in the ED. The benefit of improving triage protocols was exemplified by the 4% decrease in ESI 2 patients in the Vertical Zone and the 16% decrease in ESI 3 patients in the Fast Track.

Initially strict triage criteria resulted in intermittent periods where there were no Fast Track patients, contributing to the low 1.7 patients per hour in 2014. In 2015, Fast Track exclusion criteria were eased and hourly volume increased to 1.9, although there were still lulls in patient volume. The physical spaces of both the Vertical Zone and the Fast Track were changed multiple times over the study period due to surge protocols. The result was recurrent updates to workflows. Despite these changes, the core patient care delivery model remained constant in both treatment zones.

## CONCLUSION

The use of medical scribes to facilitate patient flow appears to be beneficial to patient throughput in a mid-acuity setting. In low-acuity zones, medical scribes were not shown to be effective at improving patient throughput likely due to faster patient turnover that requires the scribes to focus on documentation.

## Figures and Tables

**Table 1:** Patient volume, demographics, and acuity in the Vertical Zone and Fast Track.

Variables	Vertical Zone				Fast Track			

	Pre-implementation (2014)	Post-implementation (2015)	Change (%)	P-value	Pre-implementation (2014)	Post-implementation (2015)	Change (%)	P-value
Total visits, N (%)								
Discharged	3834 (73.7%)	4204 (73.4)	9.7%		3639 (98.4%)	4133 (98.2%)	13.6%	
Admitted	1266 (24.3%)	1436 (25.1)	13.4%		30 (0.8%)	54 (1.3%)	80.0%	
Total†	5201	5728	10.1%		3699	4207	13.7%	
Volume visits/day [/hour]								
Discharged	20.8 [1.6]	22.9 [1.8]	10.1%		19.8 [1.7]	22.6 [1.9]	14.1%	
Admitted	6.9 [0.5]	7.8 [0.6]	13.0%		0.1 [0]	0.3 [0]	-	
Total†	28.3 [2.2]	31.1 [2.4]	9.9%		20.1 [1.7]	23 [1.9]	14.4%	
Gender, N (%)				0.06				0.984
Male	2217 (42.6%)	2327 (40.6%)	−2.0%		1828 (49.4%)	2080 (49.4)	0%	
Age (years), N (%)				0.48				0.003
<18	70 (1.3%)	48 (0.8%)	−0.5%		1108 (30.0%)	1377 (32.7%)	2.7%	
18–64	3835 (73.7%)	4257 (74.3%)	0.6%		2386 (64.5%)	2576 (61.2%)	−3.3%	
65+	1296 (24.9%)	1423 (24.8%)	−0.1%		205 (5.5%)	254 (6.0%)	0.5%	
ESI level-discharged, N (%)								
ESI 2	144 (3.8%)	92 (2.2%)	−36.1%		-	-	-	
ESI 3	3279 (85.5%)	3665 (87.2%)	11.8%		559 (15.4%)	455 (11.0%)	−18.6%	
ESI 4	396 (10.3%)	432 (10.3%)	0%		2755 (75.7%)	3272 (79.2%)	18.8%	
ESI 5	-	-	-		324 (8.9%)	405 (9.8%)	25.0%	
ESI level-admitted, N (%)								
ESI 2	271 (21.4%)	308 (21.4%)	13.7%		-	-	-	
ESI 3	980 (77.4%)	1108 (77.2%)	13.1%		22 (73.3%)	34 (63.0%)	54.5%	
ESI 4	11 (0.9%)	16 (1.1%)	45.5%		4 (13.3%)	19 (35.2%)	375.0%	
ESI 5	-	-	-		-	-	-	

† Includes encounters without triage Emergency Severity Index (ESI) labels.

**Table 2:** Length of stay (LOS) for the Vertical Zone and Fast Track patients.

	Vertical Zone							Fast Track						

	Pre-implementation (2014)		Post-implementation (2015)					Pre-implementation (2014)		Post-implementation (2015)				

Patient disposition	LOS (min)	SD	LOS (min)	SD	Δ (min)	95% CI	P-value	LOS (min)	SD	LOS (min)	SD	Δ (min)	95% CI	P-value
Discharged	235	110	223	108	−12	−17,−7	<0.0001	89	55	95	60	6	4,9	<0.0001
Admitted	225	120	228	122	3	−6,12	0.5324	226	114	253	136	27	−29,85	0.3351
Total	233	-	224	-	−8	-	<0.0001	90	-	97	-	7	-	<0.0001

*CI*, confidence interval; *LOS*, length of stay; *min*, minutes; *SD*, standard deviation.
